# An Integrated Review of Uterine Activity Monitoring for Evaluating Labor Dystocia

**DOI:** 10.1111/jmwh.13119

**Published:** 2020-06-01

**Authors:** Katherine J. Kissler, Nancy K. Lowe, Teri L. Hernandez

**Affiliations:** 1 College of Nursing, University of Colorado Anschutz Medical Center, Aurora, Colorado

**Keywords:** cesarean birth, labor: first stage, intrapartum care, normal birth, obstetric complications, quantitative research

## Abstract

**Introduction:**

Labor dystocia is the most common cause of cesarean birth in the United States, yet how dystocia develops during labor remains elusive. Uterine activity monitoring has significant potential for advancing our understanding of labor dystocia. While evaluating contraction frequency and amplitude is a common component of labor dystocia management, the literature describing the relationship between measures of uterine activity and labor dystocia is heterogeneous and has not been synthesized to identify the best methods for use in clinical investigation.

**Methods:**

We conducted a literature search for original research exploring the relationship between uterine activity and labor dystocia published between 2000 and 2019. Included articles were critically reviewed and synthesized.

**Results:**

Across 11 identified studies, investigators employed 3 different techniques for monitoring uterine activity and 9 different measures were employed. Uterine activity measures, including Montevideo units, uterine electromyography power density spectrum and sample entropy, and the fall-to-rise ratio of contraction shape, detected patterns associated with labor dystocia or cesarean birth.

**Discussion:**

The use of multiple regression with clinical covariates and a uterine activity measure increased the accuracy of predicting cesarean delivery. Uterine electromyography may be especially useful to evaluate labor dystocia phenotypes to differentiate uterine muscle fatigue from understimulation and lead to algorithms for increased precision in the diagnosis of labor dystocia and innovative approaches to treatment.

## INTRODUCTION

Curbing the rate of unnecessary cesarean births is a national health priority because cesarean births contribute significantly to maternal and newborn morbidity and mortality, health care costs, and dissatisfaction with care.^1^ Despite multidisciplinary efforts to reduce the rate of cesarean birth, the current cesarean birth rate for low-risk women (25.9%) in the United States remains above the Healthy People 2020 target of 23.9%.^1,2^ Labor dystocia is the most common cause of cesareans performed during active labor.^3^ A working group of the American College of Obstetricians and Gynecologists (ACOG) and the Society for Maternal-Fetal Medicine (SMFM) recognized that labor progresses slower than was conventionally understood and suggested a focus on refining the definition of labor dystocia for evidence-based management to address persistently high rates of cesarean birth.^4^ Accurate measurement of uterine activity has the potential to further understand variation in the pathophysiology of labor dystocia, clarify its definition, and drive innovations to treat dystocia and prevent unnecessary cesarean birth. In this article, the existing research in which uterine activity measures were used to understand labor dystocia is evaluated.

Labor dystocia is a broad term defined statistically as cervical dilation occurring at a rate slower than the 95th percentile of normal labors.^4^ Treatment of labor dystocia is limited to oxytocin augmentation followed by cesarean birth if inadequate cervical dilation persists. However, approximately 12% of women do not respond adequately to oxytocin augmentation,^5,6^ suggesting variation in the underlying pathophysiology of labor dystocia that is not well understood.

Monitoring uterine activity is a common component of both the diagnosis and management of labor dystocia. Active management of labor dystocia typically includes calculating adequacy of uterine contractions using Montevideo units (MVUs) measured via an intrauterine pressure catheter. The oxytocin dose is titrated to achieve a value for the MVUs known to be associated with labor progress.^7^ MVUs greater than 200 is commonly used as a criterion for adequate uterine activity.^4,7,8^ More nuanced features of uterine activity have been explored but have not received wide-spread attention. Measures of contraction coordination including contraction shape, uterine electrical activity, and patterns of contractions over time may be effective for identifying uterine fatigue and differentiating it from other causes of labor dystocia such as understimulation of contractions or fetal malposition. The purpose of this integrated literature review was to synthesize existing evidence from studies of uterine activity during labor dystocia with an emphasis on evaluating measures that may identify uterine muscle fatigue.

## METHODS

**Figure 1.**
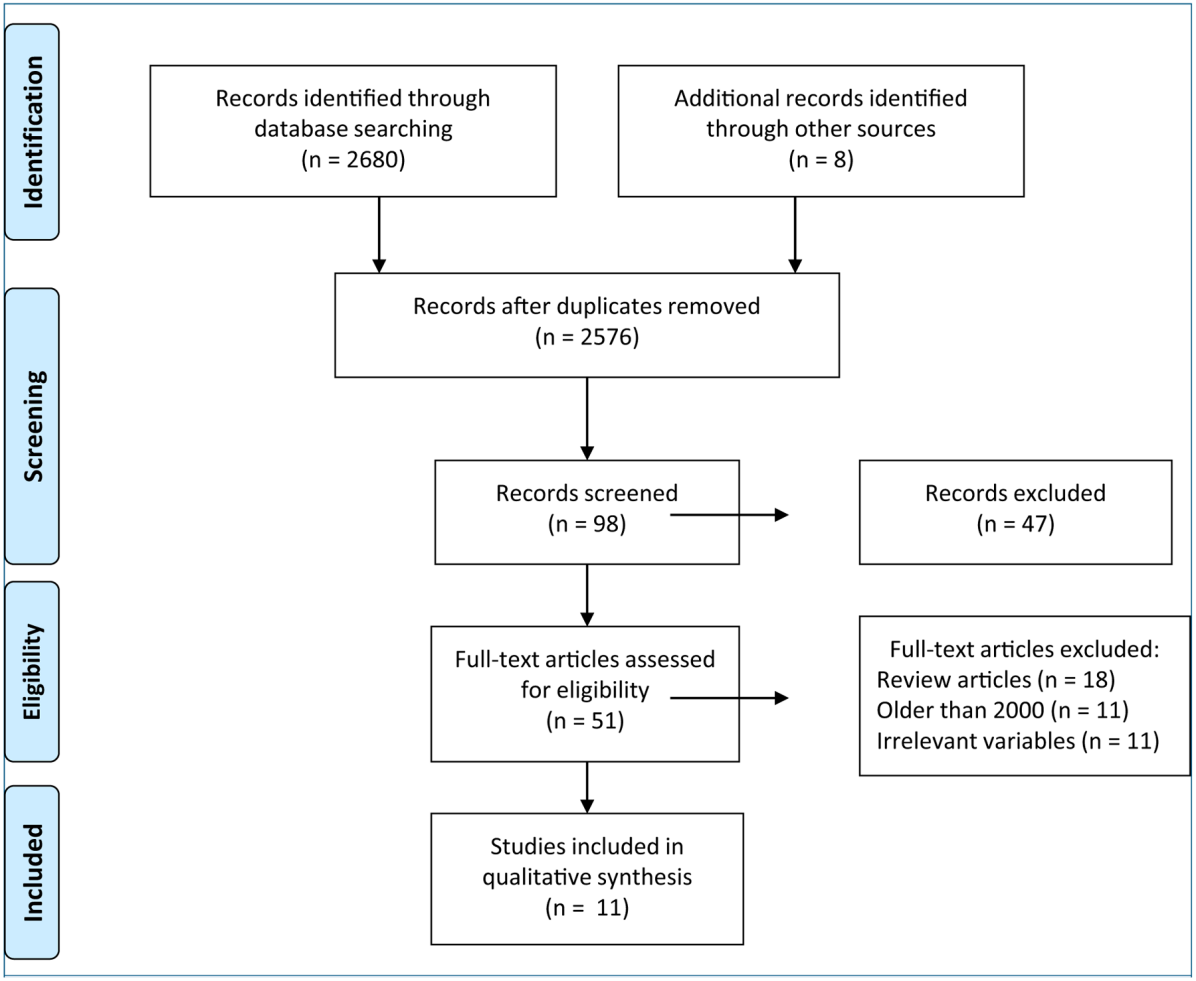
PRISMA Diagram Flow diagram of search and included studies. Source: Moher et al.^9^

A literature search was conducted in PubMed, MEDLINE, Embase, and CINAHL using a combination of search terms including *labor dystocia*, *cesarean*, *prolonged labor*, *uterine monitoring*, and *uterine contraction* from the years 2000 to 2019. English-language articles in which original research was reported that evaluated the relationship between a uterine activity measure and labor dystocia or cesarean birth were included. Studies in which any measure of labor dystocia was reported, including rate of cervical dilation, arrest of labor, and cesarean birth, were included. Articles that did not include the relevant variables, review articles, or those published prior to 2000 were excluded. After reviewing the titles, abstracts, and full text, articles were identified that met the inclusion criteria (Figure 1).^9^ The first author summarized these studies in a literature matrix that included each study’s purpose, design, sample, method of measuring uterine activity, method of measuring labor dystocia, and results (Appendix 1). All authors evaluated and synthesized the evidence. The included studies were critically analyzed with attention to both the methodology and results that were synthesized to identify current evidence supporting measurement of uterine activity and techniques that may identify labor dystocia and specifically uterine muscle fatigue.

## RESULTS

Eleven studies met inclusion criteria. The sample included papers published between 2002 and 2016 that originated in 6 countries: the United States, Canada, Slovenia, Iran, South Africa, and the Netherlands. Sample sizes ranged from 28 to 36 women in 3 smaller feasibility studies,^10–12^ 100 to 200 women in 7 observational case-control and cohort studies,^13–19^ and 503 women in one randomized controlled trial.^20^ Eight studies included only nulliparous women,^11–14,16–19^ whereas 3 included women of any parity.^10,15,20,21^

Uterine activity was measured in a variety of ways including frequency of contractions, intrauterine pressure, electrical activity measured by uterine electromyography (EMG) power density spectrum, and measures of contraction organization such as the fall-to-rise ratio, SD of contraction frequency, spatiotemporal maps, and EMG sample entropy (Table 1). Although most researchers evaluated uterine activity at a single point in time, some used multiple time points or continuous data to identify patterns of change in uterine activity during labor. The outcome variables also varied across studies including the diagnosis of labor dystocia, maternal experience of fatigue, and cesarean birth.

### Uterine Activity Measured by Temporal Indicators

Temporal measures (frequency, duration, and resting time) were common, as they were at least incidentally included in all uterine activity measurement techniques. Frequency of contractions had a mixed relationship with measures of labor dystocia in these studies. Oppenheimer et al^14^ retrospectively assessed patterns of uterine contractility in nulliparous women in spontaneous labor at term who had a cesarean for dystocia (n = 64) compared with a similar cohort who had vaginal births (n = 128). Contraction frequency was measured as the interpeak (peak-to-peak) time that represented a moving average over 5 contractions and the SD of contraction frequency measured in sequential 30-minute segments. These researchers reported that interpeak time decreased during naturally progressing labor resulting in vaginal birth. They then plotted the interpeak times against time to generate a slope that reflected contraction frequency over time. The slope was steeper for women whose contractions were more frequent over time. After augmentation, women who had labor dystocia and a vaginal birth had a greater decrease in interpeak time (postaugmentation slope, –29 s/cm; SD, 36.7) compared with those who experienced cesarean birth (postaugmentation slope, −6.2 s/cm; SD, 45.3). The significant change in contraction pattern (increased frequency) following augmentation in women who gave birth vaginally was not present in women who experienced cesarean birth, suggesting that this measure may be a tool for predicting birth route after augmentation. A similar between-group pattern was noted when the pre- and postaugmentation slopes of the SD of interpeak time were compared (preaugmentation SD slopes: normal labor, 0.8 s/cm; labor dystocia and vaginal birth, 0.4 s/cm; cesarean birth, −1.0 s/cm; postaugmentation SD slopes: labor dystocia and vaginal birth, −6.4 s/cm; cesarean birth, −0.4 s/cm). The authors reported wide variation in contraction pattern both within and between groups which limited their conclusions.^14^

In a study evaluating spatial patterns of the electrical activity of contractions, Euliano et al^10^ also measured contraction frequency with uterine EMG and reported that in women of mixed parity (N = 36), all had sustained contraction frequency of 1–3 minutes, even though 12 gave birth by cesarean for labor arrest, suggesting that frequency alone was not associated with cesarean in this small study. Ebrahimzadeh Zagami et al^17^ reported decreased mean number of contractions measured by tocodynamometry in a 30-minute period during active labor, defined as 3 to 5 cm cervical dilation, in women who experienced cesarean birth (n = 162; mean number of contractions 7.05 ± 1.46) compared with women who gave birth vaginally (n = 38; mean number of contractions 8.3 ± 2.30; *P* = .002). Findings from an earlier study by the same group indicated that contraction frequency was not significantly associated with maternal self-report of fatigue in nulliparous women in spontaneous labor with a singleton fetus in vertex position at term gestation (N = 100).^16^

Contraction duration and uterine resting time are other potentially relevant temporal measures of uterine activity; however, they were not reported in any of the identified studies. Overall, findings of the included studies suggested that decreased contraction frequency may be characteristic of a labor pattern resulting in cesarean birth, but limited precision for identifying such patterns was demonstrated. Additionally, frequency itself may not differentiate uterine fatigue from understimulation of contractions because it could be a sign of either underlying condition. However, patterns of change in contraction frequency over the course of labor and the use of frequency in conjunction with other measures may provide useful insights into the pathophysiology underlying dystocia.

### Uterine Activity Measured by Montevideo Units

MVUs are an index of uterine activity based on pressure changes (mm Hg) in the amniotic fluid during contractions measured by an intrauterine pressure catheter.^7^ Caldeyro-Barcia et al^7^ first described use of MVUs in 1957 as a tool for evaluating the pharmacologic action of oxytocin on uterine contractions. The MVU represents the product of *intensity* of contractions, characterized by the change in amplitude of intrauterine pressure (from baseline to peak) of each contraction in a designated 10-minute period, and the *frequency* of contractions, the number of contractions in that same period.^7^ In the 2014 Obstetric Care Consensus publication, ACOG and SMFM used a criterion of greater than 200 MVUs to define adequate uterine contractions in their definition of first-stage labor arrest.^4^ This criterion was based on a 1986 study of 109 women receiving exogenous oxytocin for labor induction or augmentation of labor in which 77% of women undergoing augmentation reached MVUs of 200 to 224 and 7.7% had MVUs that were higher than 300.^8^ In the more recent research included in this review, we re-examined 200 MVUs as a therapeutic goal for titrating oxytocin administration during augmentation of contractions.

Mol et al^20^ conducted a secondary analysis of data from a randomized controlled trial in which the value of intrauterine pressure catheter measurement versus external uterine monitoring was assessed in women with induced or augmented labor. Although the original study was not designed for these analyses, the authors reported that in women who gave birth vaginally (n = 403), only 47% had MVUs greater than 200 at any point during labor. The risk of cesarean birth was lower in women who had uterine activity greater than 300 MVUs (n = 76, likelihood ratio 0.41; 95% CI, 0.18–0.68). For women with uterine activity less than 100 MVUs (n = 78), the likelihood ratio of cesarean was 1.6 (95% CI, 0.98–2.5).^20^ Although the findings were not statistically significant, the authors detected a trend toward lower likelihood of cesarean birth in women who attained higher MVU values. The findings were limited by a lack of information regarding the timing of contraction data collection in relationship to the time of cesarean birth. In the parent study, the same research group reported that titration of oxytocin augmentation to uterine activity greater than 200 MVUs did not reduce the incidence of cesarean birth or adverse neonatal outcomes compared with titration to 3 to 4 contractions per 10 minutes measured by tocodynamometry.^22^

In summary, lower MVU values are associated with cesarean birth; however, because of low predictive value for cesarean birth and high frequency of women with MVUs less than 200 who have a vaginal birth, uterine activity less than 200 MVUs may not be an appropriate criterion for the diagnosis of labor dystocia. Intrauterine pressure measured at single time points is not likely to identify uterine fatigue specifically as intrauterine pressure is expected to be reduced in both fatigue and understimulation of uterine contractions.

### Uterine Activity Measured by Uterine Electromyography

**Figure 2.**
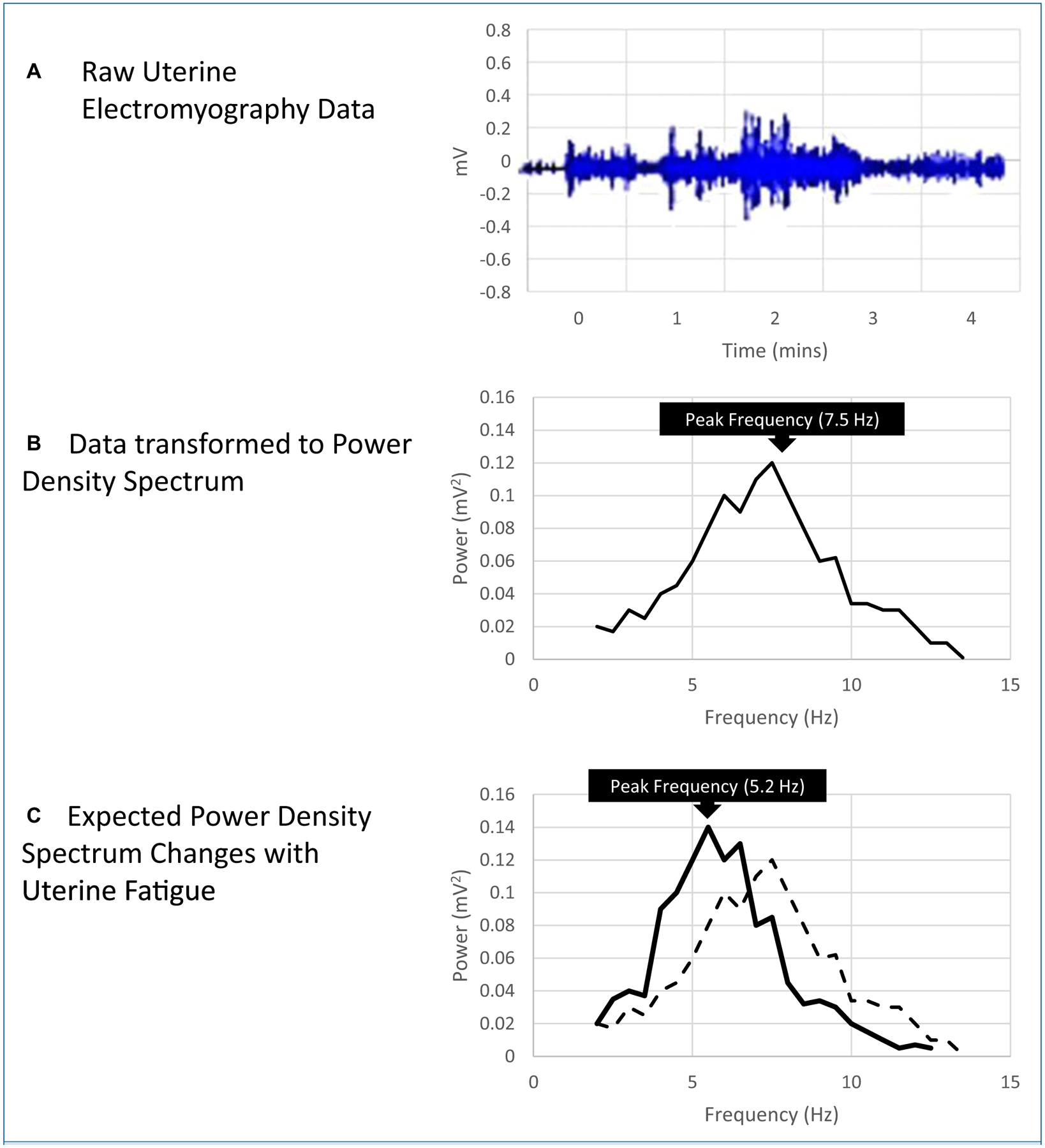
Electromyography Power Density Spectrum A) Raw electromyography (EMG) data for a single uterine contraction (electrical potential over time). B) Raw EMG transformed using Fast Fourier Transform to power density spectrum for a single uterine contraction (power over frequency). C) With uterine fatigue, the median and mean frequency of the power density spectrum is expected to decrease (left shift, x-axis) while the amplitude of the peak frequency is expected to increase (y-axis). Increased amplitude of the peak frequency (y-axis) is also associated with cesarean birth, labor dystocia, and labor augmentation. (solid line **=** fatigue; dotted line **=** normal contraction).

Uterine EMG is a measure of the electrical signaling that leads to propagation of contraction throughout the uterine muscle. The power density spectrum describes the distribution of electrical frequency components within the EMG signal. Decreases in the mean and median frequency of a power density spectrum of the EMG data accompanied by an increased peak amplitude reflect muscle fatigue prior to measurable decreases in force (Figure 2).^23^ These measures are used in exercise physiology research to evaluate skeletal muscle fatigue and are also expected to reflect uterine muscle fatigue.^23^ The peak frequency of the power density spectrum is less commonly used in exercise physiology research; however, increased peak frequency is associated with onset of labor.^24,25^

Vasak et al^13^ studied 119 nulliparous women with a term, singleton, vertex fetus who were in spontaneous labor at initiation of the study. The authors evaluated the relationship of the power density spectrum peak frequency with oxytocin augmentation and route of birth. The mean power density spectrum peak frequency was highest during labor in women who experienced cesarean birth (0.554 Hz) and lower in women who gave birth vaginally with augmentation (0.514 Hz, *P* = .01) and in those who gave birth vaginally without augmentation (0.492, *P* = .001). Women who experienced a cesarean birth had a sustained high-power density spectrum peak frequency both pre- and postaugmentation. However, women with labor dystocia who gave birth vaginally responded to augmentation with an increased power density spectrum peak frequency (preaugmentation: 0.506 Hz; 95% CI, 0.491–0.520 vs postaugmentation: 0.517 Hz; 95% CI, 0.503–0.530; *P* = .001 after Bonferroni correction).^13^ In this single study, increased mean EMG power density spectrum peak frequency was positively associated with labor dystocia, augmentation, and cesarean birth; this is contrary to hypothesized EMG changes based on shifts noted in skeletal muscle fatigue (Figure 2). The authors suggested that although increased peak frequency is generally associated with increased muscle activity, there may be a peak frequency limit above which increased peak frequency is associated with acidosis and inhibited contractility instead.^13^ This study was limited by low numbers of contractions available for analysis after the women were divided into the 3 subgroups (n = 14 gave birth by cesarean, n = 73 gave birth vaginally with augmentation, and n = 32 gave birth vaginally without augmentation).^13^

### Uterine Activity Measured by Contraction Coordination

Measures of contraction coordination or synchronization also may detect characteristics of uterine activity related to uterine fatigue with greater sensitivity than other measures. Measures of contraction coordination include fall-to-rise ratio, regularity of contraction frequency, spatial measures, and EMG sample entropy.

#### Fall-to-Rise Ratio

**Figure 3.**
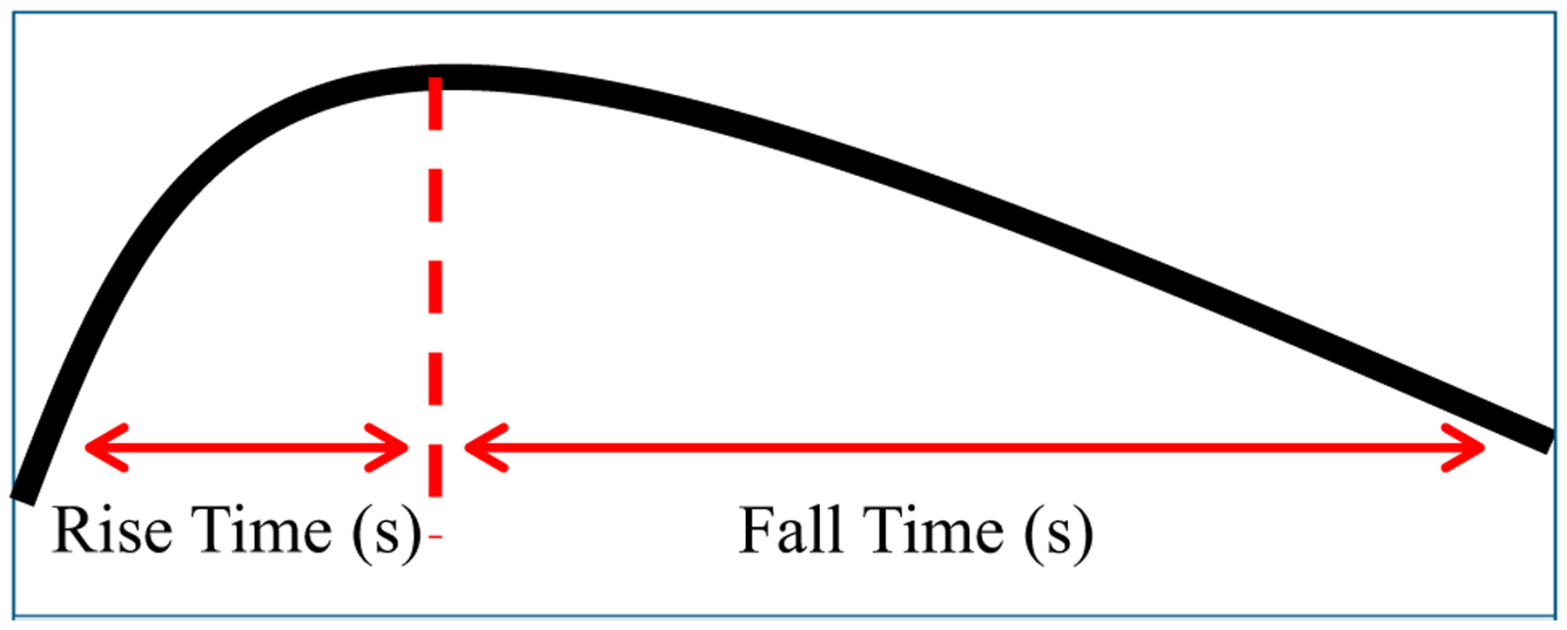
Calculating the Fall-to-Rise Ratio Fall-to-rise ratio is the time (in seconds) from the peak to the resolution of the contraction divided by the time (in seconds) from the onset to the peak of the contraction. Increased fall-to-rise ratio characterized by a prolonged recovery time following a contraction and is hypothesized to indicate uterine fatigue.

The fall-to-rise ratio is a measure of contraction shape calculated as the time in seconds from the peak of the contraction to return to baseline divided by the time from contraction onset to the contraction peak (Figure 3).^16^ The fall-to-rise ratio was reported in 4 studies,^15–18^ 3 of which were conducted by the same research group in Iran.^15–17^ Increased fall-to-rise ratio, which indicates a contraction that has a prolonged fall time compared with the rise time, was hypothesized by the researchers to indicate slow recovery of the uterine muscle following contraction. Althaus et al^18^ conducted a case-control study evaluating fall-to-rise ratio in 100 nulliparous women with a term, singleton, vertex fetus who had cesarean birth for cephalopelvic disproportion or arrest of labor matched with 100 women who gave birth vaginally. Mean (SD) fall-to-rise ratio calculated from either external tocodynamometry or intrauterine pressure monitoring was higher in women who experienced cesarean birth (1.77 [0.04]) compared with women who gave birth vaginally (1.55 [0.03], *P* = .00003).^18^ In women who received oxytocin augmentation (n = 165), the mean fall-to-rise ratio was higher in women who had a cesarean birth compared with those who had a vaginal birth (1.63 vs 1.52, *P* = .0225).^18^ The study was limited by samples within subset groups that were too small to analyze, and differences between groups were small.^18^ In a cross-sectional study, Ebrahimzadeh Zagami and colleagues^17^ corroborated the findings of Althaus et al,^18^ reporting that the mean (SD) fall-to-rise ratio calculated from tocodynamometry averaged over 30 minutes at 3 to 5 cm cervical dilatation and was higher in 38 women who gave birth by cesarean compared with 162 women who gave birth vaginally (1.64 [0.301] s vs 1.13 [0.193] s, *P* < .001). Moghaddam et al^15^ similarly found that the mean (SD) fall-to-rise ratio calculated from tocodynamometry measured over 1 hour during active labor (4–7 cm cervical dilation) was higher in 60 women who gave birth by cesarean compared with 60 women who gave birth vaginally (1.74 [0.21] vs 1.54 [0.26] respectively). However, the sensitivity (68.32%), specificity (70.01%), positive predictive value (69.55%), and negative predictive value (68.91%) of fall-to-rise ratio for cesarean birth were all low.^15^ In a biobehavioral study, Ebrahimzadeh Zagami et al^16^ reported that increased fall-to-rise ratio calculated by tocodynamometry was positively associated with women’s self-reported fatigue severity using a 100 mm visual analog scale (*r* = 0.27, *p* = .007).^16^ Taken together, these findings suggest that increased fall-to-rise ratio is associated with cesarean birth and may indicate women’s experience of fatigue. However, the fall-to-rise ratio is relatively underused, and the authors failed to evaluate reliability and validity of the measure itself.

#### Contraction Regularity

Oppenheimer et al^14^ measured the SD of the contraction frequency (the time from the peak of one contraction to the peak of the next) to evaluate contraction regularity. These researchers reported that the SD of peak uterine contraction frequency decreased over the course of normal labor (ie, contraction frequency became more regular). In women who had cesarean births, the SD of frequency decreased less (eg, contractions were less regular) than in women who gave birth vaginally (*P* = .0004).^14^

#### Spatiotemporal Pattern of Electromyography

Unlike tocodynamometry or intrauterine pressure monitoring, uterine EMG can measure the propagation of muscle contraction across the uterus, indicating the direction of force. Effective uterine contractions follow a descending pressure gradient, with the strongest and longest muscle contraction in the fundus.^10^ In a case-control study of 36 women, Euliano et al^10^ used EMG electrodes placed on the woman’s abdomen to detect different locations of electrical activity around the uterus to evaluate the fundal dominance of contractions (measured as upward movement of the center of uterine activity during a single contraction). The authors reported that fundal dominance was more common in women with normal labor and less common in women who had cesarean births for labor arrest.^10^ However, findings from a follow-up prospective cohort study (N = 167) conducted by the same research group did not support this association.^19^ Rather, the authors found that fundal dominance was not different for women experiencing cesarean birth for labor dystocia (n = 11) compared with 156 women who gave birth vaginally (proportion and SD of contractions showing fundal dominance of 88.7 [10.2] vs 86.0 [11.4], respectively; *P* = .44).^19^ Power was limited by a lower-than-expected cesarean rate (6.6%), which is significantly and substantially lower than the national rate in a similar population.^19^

In the first study, however, the researchers conducted a receiver operating curve analysis using logistic regression, which indicated that the area under the curve for predicting cesarean birth for arrest of labor using gestational age, body mass index, parity, spontaneous versus induced labor, and dilation at the time of uterine activity measurement was 79%.^10^ Adding the movement pattern of the contraction center to the equation increased the area under the curve to 91%.^10^ These findings demonstrated that addition of the covariates predicted cesarean birth with 79% accuracy and that adding fundal dominance of uterine activity further increased the predictive value.

#### Electromyographic Sample Entropy

Sample entropy is an EMG measure that evaluates coordination of contractions.^11,12^ Dysfunctional contractions are hypothesized to have disorganized electrical frequencies with greater sample entropy, reflecting more signal randomness. In 2 similar small pilot studies (N = 28 and N = 32),^11,12^ researchers demonstrated the feasibility of using sample entropy to measure dysfunctional contractions. Vrhovec et al used both uterine EMG measured with external electrode arrays and cervical EMG measured using needle electrodes inserted into cervical tissue to directly measure the muscle activity during cervical dilation.^11,12^ They reported decreased sample entropy over the course of labor (ie, increased regularity of electrical signals, suggesting effective contractions) in women who had a normal labor progress and an increase in sample entropy (ie, increased randomness of electrical signals, suggesting disorganized contractions) in women with labor dystocia.^11,12^ In women with labor dystocia, sample entropy increased during dystocia (ie, increased randomness of electrical signals) and decreased when it resolved.^11,12^

## DISCUSSION

Researchers studying labor dystocia using uterine activity monitoring have employed varied measurement techniques, research methods, and analyses. Nonetheless, authors of the studies included in our review found several measures of uterine activity that were associated with labor dystocia and cesarean birth. With regard to temporal measures, less frequent contractions were associated with labor dystocia and cesarean birth.^14,17^ The time between contractions (interpeak time) and the SD of time between contractions (ie, contraction regularity) decreased over the course of normal labor but were more likely to increase or decrease with a flatter slope in women with labor dystocia and cesarean birth.^14^ Lower MVU values were associated with cesarean birth. However, despite being a mainstay of therapy, a threshold for MVUs of less than 200 had a low predictive value for cesarean birth. Moreover, a high frequency of women had vaginal births with uterine activity less than 200 MVUs.^20,22^ Importantly, an increased fall-to-rise ratio was associated with increased incidence of cesarean birth.^15,17,18^ Similarly, increased EMG power density spectrum peak frequency^10,13^ and increased sample entropy of the EMG signal were associated with cesarean birth.^11,12^ Decreased fundal dominance of contractions measured by EMG was associated with cesarean birth in one study, but the finding was not corroborated in a follow-up study.^10^ Although many measures were associated with labor dystocia and cesarean birth, no single method was reported to have a high predictive value for mode of birth.

Our findings revealed some significant limitations of the existing published research, including heterogeneity of research methods, lack of validity and reliability data for the measurement techniques, the indirect nature of measures, and a lack of incorporation of important covariates in predicting outcomes or determining differences between groups. Most research groups developed novel methods for monitoring uterine contractions, and there currently is no consensus about how to best measure contraction features. Inconsistency across study methods makes the findings difficult to compare and synthesize into cohesive conclusions. Authors of the identified studies failed to describe reliability statistics for their own data and to establish validity of the measure for evaluating labor dystocia either theoretically or through comparison of simultaneous measures. Furthermore, researchers did not include the use of multiple measures or duplicate measurements in their study designs to evaluate reliability.

Importantly, none of the measurement techniques in the included studies directly measure the action of the uterine corpus with regard to how it affects dilation of the cervix and birth of the fetus. Rather, these measures act as proxies based on the assumption that they are measuring the effectiveness of uterine contractions. The action of the muscles of the uterine corpus on the cervix is complicated and consists of both increasing the pressure on the fetus and amniotic fluid to produce a downward force against the cervix and pulling the cervix upward toward the fundus.^26^

There are a myriad of variables that may contribute to labor dystocia. Demographic factors such as maternal age and clinical factors such as body mass index, fetal positioning, gestational age, estimated fetal weight, epidural analgesia use, and induction of labor all may contribute to labor dystocia and subsequent medical or surgical interventions. Increased maternal age and obesity, 2 increasingly prevalent factors among childbearing women, increase the risk for labor dystocia because of their presumed effects on uterine contractility.^27–31^ Based on existing research, it is not clear whether age, obesity, and clinical factors confound, mediate, or moderate the relationship between uterine activity and labor dystocia. Whenever possible, women with obesity and of advanced maternal age should be included in research and other covariates considered in analyses to evaluate measures of uterine activity and relationships with outcome variables, including labor dystocia and cesarean birth to appreciate how these variables contribute to the pathophysiology of labor dystocia. Multiple regression modeling, such as that conducted by Euliano et al,^10^ may be especially useful. With multiple clinical covariates, the area under the curve of the receiver operating curve for identifying cesarean was .79, but addition of a uterine activity measure (fundal dominance) increased the area under the curve to .91. Thus, uterine activity measures may add value to other clinical factors in research aimed at understanding labor dystocia.

Making multiple or continuous measurements over the course of labor is an effective way to account for individual variation and identify patterns in both individual and aggregate data. Analyzing patterns of contraction change controls for interindividual variation in contraction characteristics. Multilevel modeling could also be used to evaluate change over time within and between individuals. Although Mol et al^20^ and Vasak et al^13^ used data that were collected at multiple time points during labor, they summed or averaged the data rather than analyzing them longitudinally. In contrast, Vrhovec^11^ and Oppenheimer et al^14^ reported contraction data longitudinally and identified distinct patterns of change in uterine contractions during normal and abnormal labor. Modeling covariates and patterns over the course of labor may further allow for targeted evaluation of uterine fatigue. Differentiating the relationships of covariates with uterine fatigue from those with understimulation of contractions may reveal the mechanisms by which obesity, age, and other covariates affect the pathophysiology of labor dystocia. In addition, modeling patterns of uterine activity measured over the course of labor may help to reveal the underlying pathophysiology. The nature of how a measure changes over time may be more meaningful than the measure at a single time point.

## CONCLUSION

Findings from the studies reviewed on uterine activity monitoring demonstrate that existing techniques and measures have potential for contributing to research aimed to advance understanding of variation in the pathophysiology of labor dystocia. There is a need for additional research to determine which contraction markers most accurately reflect labor dystocia, differentiate the pathophysiology of types of labor dystocia, predict responsiveness to intervention, and ultimately determine whether interventions based on contraction monitoring can be effective in improving outcomes.

## Figures and Tables

**Table 1.** List of Uterine Activity Monitoring Techniques, Measurements, Definitions of Measurements, and Units

Type of Uterine Activity Monitor	Most Common Measurements	Definition and Units of Measurement	Characteristics of Uterine Activity Monitor
Tocodynamometry Pressure exerted by the external maternal abdomen against a piston within a transducer	Contraction frequency	Number of contractions in 10 or 30 min	Tracing can be used to calculate contraction frequency, duration, and resting time between contractions
	Peak-to-peak time	Time in sec from the peak of one contraction to the peak of the next contraction averaged over 30 min	
			Recorded amplitude is relative rather than absolute
			Considered noninvasive, although women report that it is uncomfortable and may limit mobility
	Fall-to-rise ratio	Time in sec from the peak of a contraction until the return to baseline divided by the time in sec from the onset of the contraction until the peak	
			Can identify tachysystole and timing of decelerations in the fetal heart rate
	SD of peak-to-peak time	SD of peak-to-peak time of each contraction over 30 min	
Intrauterine pressure fluid (pressure within an intrauterine pressure catheter inserted into the uterine cavity around the presenting part of the fetus)	Contraction frequency	Number of contractions in 10 or 30 min	Considered the gold standard for clinical uterine monitoring especially during labor dystocia
			Tracing can be used to calculate contraction frequency, duration, amplitude, resting time, and resting pressure
	Montevideo units	The sum of the change in amplitude of each contraction (peak pressure minus the baseline pressure) over 10 min	Amplitude is considered absolute
			Considered invasive and requiring rupture of membranes prior to placement
Uterine EMG Differentials in electrical potential across uterine muscle using an electrode array placed on the maternal abdomen. Signals are filtered and processed to provide various measures	EMG power density spectrum mean, median, and mode (peak)	Measured over an identified period of time in frequency (Hz)	Processed tracing can be used to calculate contraction frequency, duration, and resting time between contractions
		Reported as movement of uterine electrical as either toward the fundus or toward the lower uterine segment activity during the rise and fall of the contraction	
	Spatiotemporal maps		
	Sample entropy		Does not measure amplitude
			Additional processing and mathematical transformation provide information about the electrical frequencies hypothesized to identify uterine muscle fatigue
		Nonparametric measure of entropy in uterine EMG	
			Considered noninvasive, although women report that it can be uncomfortable and limit mobility

Abbreviations: EMG, electromyography

## Quick Points

- Existing clinical and research-focused uterine activity measures are associated with labor dystocia and cesarean birth; however, no measure is highly sensitive or specific for predicting outcomes in labor dystocia.
- Uterine electromyography is sensitive to muscle fatigue and may present a unique technique for identifying uterine muscle fatigue contributing to labor dystocia.
- Additional research is needed to determine which contraction measures most accurately reflect labor dystocia, differentiate the pathophysiology of types of labor dystocia, predict responsiveness to intervention, and ultimately determine whether contraction monitoring improves outcomes.

## Appendix 1

**Table:** Review Matrix for Extant Literature on Contraction Activity Measures and Labor Dystocia

Primary Author	Study Description	Results
Althaus et al^18^	Purpose to evaluate the association between F:R and cephalopelvic disproportion.	Increased F:R was associated with cesarean for labor arrest (F:R, 1.55; SD, 0.28; 95% CI, 0.91–2.30 for vaginal birth vs 1.77; SD, 0.40; 95% CI, 1.0–2.46 for cesarean; P = .00003).
	Retrospective, case-control study of nulliparous women with a single, term fetus in the vertex position in the United States (n = 200, 100 with cephalopelvic disproportion and 100 without).	
		In women who received oxytocin. augmentation, increased F:R was associated with cesarean (F:R, 1.52 for vaginal birth vs 1.63 for cesarean; P = .0225).
	Uterine contractions monitored for 1 h by external TOCO or intrauterine pressure at ≥4 cm dilatation.	
	F:R calculated for each contraction and averaged over one hr.	
Ebrahimzadeh et al^16^	Purpose to assess the correlation between self-reported maternal fatigue and F:R.	Increased F:R was associated with fatigue severity (r = 0.27; P = .007).
	Cross-sectional study in Iran (n = 100 nulliparous women).	Length of first stage of labor was also associated with fatigue severity although less strongly.
	F:R of each contraction measured by TOCO averaged over 30 min.	
		Contraction frequency was not significantly associated with fatigue severity.
	Self-report fatigue was assessed at 3–4 cm dilation using a visual analog scale anchored with “no fatigue at all” to “the most fatigue I’ve ever had.”	
Ebrahimzadeh Zagami et al^17^	Purpose to evaluate the relationship between F:R and cesarean for labor dystocia.	Increased mean F:R was associated with cesarean (F:R 1.13, SD 0.193 for vaginal birth vs 1.64, SD 0.30 seconds for cesarean, P < .001).
	Cross-sectional prospective study of nulliparous women in spontaneous labor with a term, singleton, vertex fetus in Iran (n = 200, 162 with vaginal birth and 38 with cesarean).	
		Decreased frequency of contractions was associated with cesarean (F:R, 8.3; SD, 2.3 for vaginal birth vs 7.05; SD, 1.46 for cesarean; t = 3.192; P = .002).
	F:R measured by TOCO measured at 3–5 cm dilation and averaged over 30 min.	
	Frequency of contractions was measured as the number of contractions in 30 min.	Newborn weight, height and head circumference were all significantly higher in the cesarean group (P < .001, P = .001, P = .003, respectively).
Euliano et al^10^	Purpose to compare spatiotemporal patterns of uterine electrical activity in normal and arrested labors.	All participants sustained contraction frequency of every 1–3mins and of the 11 women with cesarean, all achieved MVUs >150 mm Hg.
	Case-control study using data derived from a previous study of women in spontaneous labor in the United States (n = 36, 12 women with cesarean for active-phase arrest matched to 24 women in a control group with vaginal birth).	
		Predominately upward fundal movement was more common in those with vaginal birth (23/24 with vaginal birth vs 4/12 cesarean, P = .003).
	Using a 30-min uterine EMG segment during arrest or at the same dilation in women in the control group, the center of uterine electrical activity was identified, and the vertical motion of the center determined for each contraction.	ROC analysis of logistic regression model including gestational age, BMI, parity, spontaneous vs induced labor and dilation at time of measurement gave an AUC of 0.91 for predicting outcome based on the different patterns of movement.
Edwards et al^19^	Purpose to evaluate the relationship between fundal dominance of uterine electrohysterography and cesarean birth for labor dystocia.	Fundal-dominant contractions were not associated with cesarean for labor dystocia (88.7% ± 10.2 with cesarean for labor dystocia vs 86.0% ± 11.4, *P =* 0.44).
	Prospective cohort study of nulliparous women at term in spontaneous labor (n = 167, 11 with cesarean for labor dystocia, n = 156 all others).	
Moghaddam et al^15^	Purpose to find a correlation between F:R and cesarean for failure to progress or cephalopelvic disproportion.	Increased F:R was associated with cesarean (F:R, 1.54; SD, 0.26 for vaginal birth vs 1.74; SD, 0.21 for cesarean; OR = 0.44; 95% CI, 0.005–0.42; P < .001).
	Prospective cohort study of women in labor in Iran (n = 120, 60 with vaginal birth and 60 with cesarean for failure to progress or cephalopelvic disproportion).	
		Sensitivity = 68.32%, Specificity = 70.01%, Positive predictive value = 69.55%, negative predictive value = 68.91%.
	F:R measured by TOCO at 4–7 cm dilation, without oxytocin augmentation, for each contraction and averaged over 1 h.	ROC analysis AUC .75 with a cutoff point of 1.68.
		Increased F:R associated with maternal age (r = 0.19, P < .001).
Mol et al^20^	Purpose to provide insight in the lack of a positive effect of IUPC vs TOCO by evaluating the MVU in correlation with dysfunctional labor and adverse neonatal outcome.	Only 47% of women with vaginal birth reached MVU >200.
		Risk of cesarean was higher in women who had lower MVUs during labor (likelihood ratio 1.6; 95% CI, 0.98–2.5 for MVU <100 vs 0.41; 95% CI, 0.18–0.68 for MVU >300).
	Secondary analysis of a randomized controlled trial of women with IUPC monitoring in the Netherlands (n = 503, 403 with vaginal birth and 100 with cesarean).	
		MVUs were not associated with adverse neonatal outcomes.
	Highest MVU measured at any time in labor categorized as <100, 100–199, 200–299, or ≥300.	Lower MVUs were associated with older women, longer gestational age, longer labors, and higher birth weight.
	MVUs were also measured at last vaginal examination during the first stage of labor and categorized as <200 or >200.	
Oppenheimer et al^14^	Purpose to evaluate the relationship of the slopes of contraction frequency and SD of contraction frequency over the course of labor with augmentation and cesarean.	The interpeak interval decreased over the course of normal labor and the slope was steeper than in women with labor dystocia and/or cesarean (mean slope of interpeak frequency, −47.15 with vaginal birth and no augmentation; mean slope of interpeak frequency, −6.15 with cesarean following augmentation, P = .0004).
	Case-control study of women in labor in Canada (n = 192, 64 with cesarean matched with 128 with vaginal birth).	
	TOCO reviewed retrospectively in successive 30-min periods between 3 and 8 cm dilation	
		SD of interpeak interval followed a similar pattern as the interpeak interval (mean slope of SD, −0.76 with vaginal birth and no augmentation; mean slope of SD, +0.4, with cesarean following augmentation)
	Frequency was calculated as the average of 5 consecutive interpeak intervals.	
	SD of 5 consecutive interpeak intervals was recorded.	
Vasak et al^13^	Purpose to evaluate whether uterine EMG identifies inefficient contractions leading to arrest of labor and cesarean.	Mean power density spectrum peak frequency was higher in women with cesarean for first-stage labor arrest following augmentation than in women giving birth vaginally without augmentation or with augmentation (P = .001 and .01, respectively).
	Cohort study of n = 119 nulliparous women with a term, singleton, vertex fetus in spontaneous labor in the Netherlands (n = 119, 32 with vaginal birth without augmentation, 73 with vaginal birth following augmentation, 14 with cesarean following augmentation).	
		In women who gave birth vaginally with augmentation, mean power density spectrum peak frequency was higher after augmentation (P = .001).
	Uterine EMG power density spectrum peak frequency was calculated and averaged throughout admission.	
Vrhovec et al^12^	Purpose to evaluate the relationship between EMG sample entropy and labor progress.	In normally progressing labor, sample entropy decreased from median 0.15 (range, 0.13–0.25) at 3 cm dilation to median 0.09 (range, 0.08–0.11) at birth measured on the abdominal surface and from median 0.12 (range, 0.08–0.13) at 3 cm to median 0.03 (range, 0.03–0.05) measured from the uterine corpus. With labor dystocia, only the cervix EMG was reported. Sample entropy decreased during periods of normal progression and increased during dystocia. Median sample entropy was 0.14 (range, 0.08–0.15) at 3 cm, median 0.05 (range, 0.02–0.13) prior to the delay, median 0.22 (range, 0.18–0.25) during the delay, and median 0.04 (range, 0.02–0.05) when normal progress resumed.
	Cohort study of nulliparous women with a term, singleton, vertex fetus in induced labor in Slovenia (n = 28).	
	EMG was collected throughout hospital admission using an electrode array consisting of 12 bipolar electrodes on the abdominal surface and cervix.	
	Sample entropy was calculated in contraction bursts and successive nonoverlapping segments.	
	Labor progress, based on fetal head station was graphed on the partogram, and classified as delayed when station maintained the same value for 2 hr or more.	
Vrhovec^11^	Purpose to evaluate feasibility of measuring labor dystocia with sample entropy calculated from uterine EMG.	In women with normal labor (n = 13), sample entropy decreased throughout labor to birth.
	Prospective cohort study of nulliparous women with a term, singleton, vertex fetus in labor in Slovenia (n = 32, 13 with normal labor, 4 with delayed labor, 15 with augmented labor).	In delayed labors (n = 4), sample entropy increased during the delay and then continued to decrease.
		In augmented labor (n = 15), sample entropy increased during slow labor progress, decreased through periods of oxytocin augmentation, and increased with every additional slowing in labor progress, prior to increasing the dose of oxytocin.
	Contractions were measured throughout admission for labor by EMG using 12 bipolar electrodes on the abdominal surface and/or a cervical probe.	
	Sample entropy was calculated in successive nonoverlapping segments and graphed over time along with cervical dilation and head station.	

Abbreviations: AUC, area under the curve; BMI, body mass index; EMG, electromyography; F:R, fall-to-rise ratio; IUPC, intrauterine pressure catheter; MVU, Montevideo unit; OR, odds ratio; ROC, receiver operator characteristic curve; TOCO, tocodynamometry.
